# Therapeutic Approaches for Treating Pulmonary Arterial Hypertension by Correcting Imbalanced TGF-β Superfamily Signaling

**DOI:** 10.3389/fmed.2021.814222

**Published:** 2022-01-24

**Authors:** Patrick Andre, Sachindra R. Joshi, Steven D. Briscoe, Mark J. Alexander, Gang Li, Ravindra Kumar

**Affiliations:** Discovery Group, Acceleron Pharma (a wholly-owned subsidiary of Merck & Co., Inc.), Cambridge, MA, United States

**Keywords:** activin A, SMAD1/5/8, SMAD2/3, PAH, TGF-β, BMP, BMPRII, cell proliferation

## Abstract

Pulmonary arterial hypertension (PAH) is a rare disease characterized by high blood pressure in the pulmonary circulation driven by pathological remodeling of distal pulmonary arteries, leading typically to death by right ventricular failure. Available treatments improve physical activity and slow disease progression, but they act primarily as vasodilators and have limited effects on the biological cause of the disease—the uncontrolled proliferation of vascular endothelial and smooth muscle cells. Imbalanced signaling by the transforming growth factor-β (TGF-β) superfamily contributes extensively to dysregulated vascular cell proliferation in PAH, with overactive pro-proliferative SMAD2/3 signaling occurring alongside deficient anti-proliferative SMAD1/5/8 signaling. We review the TGF-β superfamily mechanisms underlying PAH pathogenesis, superfamily interactions with inflammation and mechanobiological forces, and therapeutic strategies under development that aim to restore SMAD signaling balance in the diseased pulmonary arterial vessels. These strategies could potentially reverse pulmonary arterial remodeling in PAH by targeting causative mechanisms and therefore hold significant promise for the PAH patient population.

## TGF-β Superfamily Dysregulation Is a Critical Component of PAH

In pulmonary arterial hypertension (PAH), pathologic vascular remodeling distorts the gross- and micro-scale structure of the pulmonary arterial vasculature, severely disrupting blood flow patterns throughout the cardiopulmonary circulation. The primary pathology is thought to originate in the small distal arterioles, in which uncontrolled proliferation of vascular cells results in narrowing and occlusion of the vascular lumen. Loss of luminal space in turn increases pulmonary vascular resistance and pulmonary arterial pressure, leading to strain on the right cardiac ventricle and ultimately to heart failure (1).

**Figure 1:**
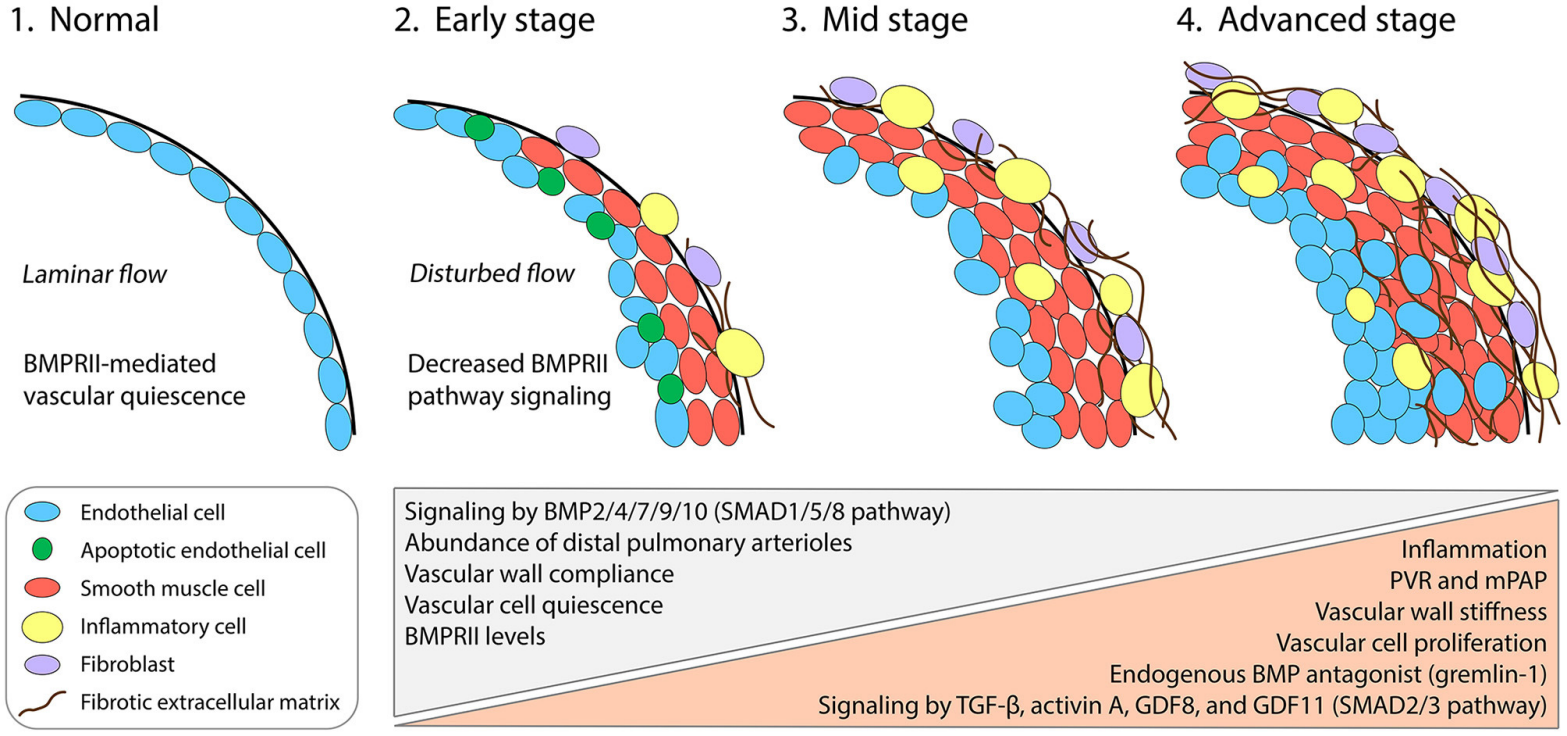
Cellular, molecular, and biomechanical progression of PAH in the pulmonary arterial wall. Under normal conditions, the pulmonary distal arterioles comprise an intimal monolayer of ECs and are largely devoid of a medial layer with SMCs. Laminar blood flow patterns promote BMPRII-pathway signaling in ECs and maintain vascular quiescence (1). Genetic mutations or combined insults lead to insufficient BMPRII levels and SMAD1/5/8 signaling in multiple vascular cell types. Early events during PAH pathogenesis include the onset of vascular wall stiffening and inflammatory responses, including infiltration by diverse inflammatory cell types. Apoptotic ECs appear early but are progressively replaced by apoptosis-resistant and hyperproliferative ECs, which ultimately form disorganized neointimal lesions (2–4). SMAD2/3 pathway-activating ligands including TGF-β, activin A, GDF8, and GDF11 become upregulated and contribute to arterial remodeling. Gremlin-1, a key pathogenic protein in PAH, reduces BMPRII-pathway signaling by antagonizing specific BMPs. SMCs accumulate in the medial layer, causing distal muscularization. Fibroblasts in the adventitial layer become activated and synthesize fibrotic extracellular matrix. PVR, pulmonary vascular resistance; mPAP, mean pulmonary arterial pressure.

Multiple cell types of the pulmonary arterial wall contribute to vascular remodeling in PAH (Figure 1) (2). Smooth muscle cells (SMCs) over-proliferate and thereby thicken vessel walls and cause vascular muscularization, including around the distal arterioles where SMCs are not normally found. Endothelial cells (ECs) also over-proliferate and in later stages of disease can form neointimal lesions that obstruct distal arterioles (3). Accordingly, targeting the proliferation of SMCs and ECs to treat PAH has been the subject of extensive efforts over the last two decades. Studies on the platelet-derived growth factor receptor pathway, which is strongly upregulated in the distal pulmonary arteries of PAH patients and contributes to over-proliferation (4), have suggested that reversal of pathology is clinically achievable (5, 6)—although safer alternative strategies are desirable (7). Research into PAH disease mechanisms has also highlighted the critical roles of other signal transduction pathways, especially those of the transforming growth factor-β (TGF-β) superfamily, which interact with inflammatory processes and biomechanical forces to regulate EC and SMC proliferation.

The TGF-β superfamily features more than 30 ligands, which together regulate a great variety of developmental and homeostatic processes in all major organs including the vasculature (8). Indeed, dysregulation of TGF-β superfamily signaling has been implicated in numerous cardiomyopathies and vasculopathies, including atherosclerosis, vascular calcification, Marfan syndrome, Loeys-Dietz syndrome, and hereditary hemorrhagic telangiectasia, in addition to PAH (9–12). Typically, binding of a dimeric TGF-β superfamily ligand promotes assembly of a heterotetrameric signaling complex comprising two type I and two type II receptor serine/threonine kinases. Upon ligand binding, the constitutively active type II receptor phosphorylates the type I receptor, activating the type I receptor intracellular kinase domain. Signal is then propagated through various canonical (involving SMAD transcription factors) and non-canonical (or SMAD-independent) pathways. In PAH, recent evidence indicates a signaling imbalance between the two principal canonical pathways, with underactive SMAD1/5/8 signaling occurring alongside overactive SMAD2/3 signaling in pulmonary arterial ECs and SMCs (13). In the remaining sections, we describe how this SMAD signaling imbalance influences the exuberant cell proliferation underlying vascular remodeling and describe therapeutic approaches for either attenuating excessive SMAD2/3 signaling or restoring deficient SMAD1/5/8 signaling in diseased pulmonary vasculature (14–20). The potential involvement of non-canonical TGF-β superfamily pathways in PAH pathogenesis is poorly understood, but these signaling mechanisms have been implicated in related pathological conditions, such as fibrosis, and merit further study. We therefore refer the reader to previous reviews covering non-canonical signaling in disease (21–25).

## Deficient Signaling in Anti-Proliferative SMAD1/5/8 Pathway

Studies exploring the human genetics of PAH have revealed important insights into PAH pathobiological mechanisms. Mutations in *BMPR2*, which encodes bone morphogenetic protein receptor type II (BMPRII), were discovered in 2000 as the first known genetic cause of PAH (26, 27). Such mutations account for >70% of inherited PAH cases and 20% of spontaneous cases, by far the largest proportion for any single gene locus (28–30). Reduced levels of BMPRII protein have been found in other forms and etiologies of PAH, even in the absence of *BMPR2* mutation, suggesting that this signaling pathway could be a point of convergence among multiple distinct PAH disease etiologies (31–33).

BMPRII pathway activity is important in both pulmonary arterial ECs and SMCs, although the two vascular cell types appear to depend on different BMPRII ligands and on different BMPRII signaling outputs. Circulating bone morphogenetic protein 9 (BMP9) and BMP10 are thought to be critical quiescence factors in the pulmonary arteries and act primarily upon ECs (34). Mice with *Bmpr2* ablated selectively in ECs develop PAH-like disease, including proliferating ECs and SMCs, highlighting the importance of BMPRII signaling in the endothelium in particular (35). In addition, BMPRII-deficient human ECs in culture undergo enhanced transformation to a proliferative and synthetic mesenchymal phenotype, suggesting that BMPRII-mediated signaling in the endothelium preserves vascular structure by promoting EC quiescence (36, 37). In contrast, BMP2 and BMP7 promote SMC apoptosis through BMPRII (38), and BMP4 reduces SMC proliferation (39, 40). Thus, loss of BMPRII from SMCs could decrease BMP2/4/7 signaling and result in the accumulation of apoptosis-resistant and hyperproliferative SMCs—hallmarks of distal arterial muscularization in PAH. Gremlin-1, an endogenous antagonist of BMP2/4/7, is markedly upregulated in PAH (Figure 1) (41, 42), which could potentially account for reduced BMPRII pathway activity in patients with normal *BMPR2* expression. Notably, recent evidence indicates that BMPRII-deficient macrophages are also important contributors to vascular remodeling in PAH, underscoring the complexity of PAH pathogenesis and the interactions between vascular cell types of different lineages (43).

Interestingly, mutations associated with PAH have also been discovered for several TGF-β superfamily members that interact functionally with BMPRII in pulmonary ECs and SMCs (44, 45). Additional PAH risk genes include those encoding the BMPRII ligands BMP9 and BMP10 (46–48); the BMPRII signaling partners activin receptor-like kinase 1 (ALK1) and endoglin (49–51); the BMPRII transcriptional mediators SMAD1, SMAD4, and SMAD8 (52, 53); and the scaffolding protein caveolin-1 (54–56), which regulates BMPRII signaling through its localization and internalization. Although not all PAH risk factors are associated with BMPRII function, the striking enrichment for TGF-β superfamily members clearly identifies the BMPRII signaling axis as a pathway necessary for pulmonary vascular homeostasis (44). Together, evidence stemming from human genetics and preclinical experiments suggests that the BMP-BMPRII-SMAD1/5/8 pathway performs a protective function and is necessary to prevent vascular cell proliferation and consequent pathologic vascular remodeling (Figure 1). However, it is important to note that experiments *in vitro* reveal that BMPRII-deficient ECs could gain SMAD1/5 responsivity to TGF-β through lateral signaling (36), suggesting possible additional levels of signaling complexity in a tissue context. It will therefore be important to resolve the states of SMAD1/5/8 and SMAD2/3 phosphorylation in a cell type–specific manner in the lungs of PAH patients.

## Overactive Signaling in Pro-Proliferative SMAD2/3 Pathway

Whereas the BMPs signal predominantly through the SMAD1/5/8 canonical pathway, other TGF-β superfamily members, notably TGF-β and the activin-class ligands, instead signal mainly through SMAD2/3. Recent evidence has revealed pathogenic roles for multiple SMAD2/3 pathway-activating ligands in PAH vascular remodeling and in the control of vascular cell proliferation (13, 57), providing important new targets for therapeutic development.

In PAH patients, elevated TGF-β levels have been detected in remodeled distal arterioles and in the circulation (Figure 1) (58–60). TGF-β can inhibit apoptosis of SMCs through activation of a non-canonical PI3K/AKT pathway and can promote SMC proliferation through a non-canonical PTEN-dependent pathway (61, 62). Blockade of signaling by one or more TGF-β isoforms using a soluble ligand trap (57), a pan–TGF-β antibody (63), or a TGF-β receptor antibody (64) demonstrates that TGF-β signaling plays a direct role in vascular remodeling and narrowing. Systemic administration of a TGF-β ligand trap decreases phosphorylated SMAD2 in the lungs of a PAH rat model, suggesting that TGF-β exerts at least some of its remodeling effects through canonical signaling in addition to non-canonical pathways (57). Beyond its direct effects in vascular remodeling, TGF-β also induces expression of endothelin-1 (ET-1) by ECs, an additional pathogenic factor in PAH (65). Increased levels of ET-1 reduce BMPRII expression (66), and BMPRII knockdown increases ET-1 (67), suggesting that a positive feedback loop could link diminished BMPRII output with enhanced signaling by TGF-β and ET-1 during PAH pathogenesis.

Activin-class ligands, which include activin A, growth differentiation factor 8 (GDF8), and GDF11, have more recently been implicated in PAH pathogenesis (13). These ligands, like TGF-β isoforms, activate SMAD2/3 signaling and might therefore act in concert with TGF-β, exerting pathogenic effects through overlapping or distinct mechanisms during pathologic vascular remodeling in PAH. Immunohistochemical evidence indicates that activin A, GDF8, and GDF11 are upregulated in small pulmonary arteries of PAH patients and PAH rodent models (Figure 1) (13). As we describe further below, concurrent inhibition of multiple activin-class ligands imparts robust protection in PAH rodent models and in phase 2 clinical trials. The individual contributions made by activin A (68) and GDF11 (69) have been explored in preclinical studies. Activin A in particular appears to play a substantial pathogenic role: it is upregulated by ECs in PAH lung tissues, can perturb EC function in culture, causes BMPRII downregulation, and when overexpressed selectively in mouse ECs can cause PAH-like disease featuring muscularized pulmonary arteries and right heart hypertrophy (68). Selective ablation of *Gdf11* in mouse ECs protects against experimental PAH (69), suggesting that GDF11 might act similarly to activin A, a close phylogenic relative (70). Notably, GDF11 can signal through the type I receptor ALK5, better known as the principal SMAD2/3-activating receptor used by TGF-β (71, 72), providing a potential mechanism for convergence of GDF11- and TGF-β-mediated signals. Given emerging evidence of GDF8 involvement in vascular dysfunction and chronic inflammatory disease (11, 12, 73, 74), it will be important in future studies to dissect the pathogenic contributions made specifically by GDF8, if any, to vascular remodeling in PAH. Whether any of the activin-class ligands drive pathologic vascular remodeling processes through non-canonical signaling mechanisms has not yet been investigated to our knowledge.

In addition to their roles in pathologic vascular cell proliferation, TGF-β superfamily ligands also control the excessive deposition of extracellular matrix, or fibrosis, that leads to vascular wall stiffness in later stages of PAH progression (Figure 1) (75). TGF-β1 in particular has long been regarded as a master regulator of fibrosis, but accumulating evidence also implicates TGF-β2 and TGF-β3 isoforms in fibrotic processes potentially relevant to PAH vascular remodeling (58, 76, 77). Individually or in combination, the three TGF-β isoforms are thought to promote myofibroblast differentiation, drive the synthesis and deposition of extracellular matrix proteins, and might stimulate mesenchymal transformation of endothelial or other cell types in the pulmonary arteries (36, 78). Interestingly, lung BMPRII and phosphorylated SMAD1/5/8 levels were found to be decreased in a model of pulmonary hypertension associated with pulmonary fibrosis, suggesting that SMAD signaling balance might coordinately regulate fibrosis together with cell proliferation (79). Many important mechanisms of arterial fibrogenesis in PAH, including the potential involvement of activin-class ligands, require further study. It is clear, however, that PAH pathogenesis is characterized by multiple pathogenic ligands acting in parallel—in complex and potentially combinatorial modes—upon distinct classes of vascular cell types.

## Interplay Between Inflammation and TGF-β Superfamily Signaling in PAH

Partial disruption of pulmonary vascular BMPRII signaling is not sufficient to initiate PAH pathogenesis because only a subset of mutation carriers is thought to develop overt disease. For *BMPR2* mutation carriers, penetrance is estimated to be ~27% (14–42%) (28–30, 80, 81). As such, additional stimuli have been proposed as “second hits,” which could potentially decrease BMPRII expression or activity below a certain threshold necessary for disease. Inflammation is considered one likely candidate for a second hit in PAH (82, 83). In animal models, inflammation precedes clear evidence of structural alterations and might be a key determinant of disease onset and progression (84). Multiple classes of immune cells, including macrophages, T cells, and neutrophils, have been identified in the vicinity of remodeled pulmonary arteries of PAH patients and PAH rodent models (Figure 1) (85, 86). Furthermore, inflammatory gene signatures have been found in cardiac and pulmonary tissues from patients and animal models of PAH (87).

Multiple lines of evidence indicate a close relationship between inflammation and BMPRII pathway signaling during PAH pathogenesis. For example, mice heterozygous for a *Bmpr2* null allele, but not wild-type controls, become more likely to develop PAH-like disease when overexpressing 5-lipoxygenase, which causes a sustained inflammatory response (88). Similarly, *Bmpr2* haploinsufficient rats are more prone to inflammation-induced PAH and exhibit evidence of apoptosis-resistant and proliferative ECs and enhanced mesenchymal transformation (89). Impaired BMPRII activity is also associated with pulmonary overexpression of inflammatory mediators including interleukin-6 (IL-6) and granulocyte-macrophage colony-stimulating factor, which are involved in leukocyte recruitment and PAH pathogenesis (90–92). Finally, mice with *Bmpr2* ablated from monocyte-lineage macrophages exhibited muscularized pulmonary arteries and increased right ventricular systolic pressure after Sugen-hypoxia treatment while depletion of macrophages with clodronate reversed these parameters (43). Together, these studies suggest that BMPRII-mediated signaling within the pulmonary vasculature normally protects against inflammation-induced vascular remodeling.

If levels of BMPRII activity become deficient, then otherwise innocuous inflammatory signals could initiate a feed-forward loop of pathological signaling by TGF-β (36), activin-class ligands (13), and other proinflammatory cytokines (43). IL-6 is a key inflammatory signal upregulated in the serum and lungs of patients with PAH (93). Transgenic mice overexpressing IL-6 in the lungs exhibit pulmonary arterial muscularization and proliferative arteriopathy, indicating that this molecule regulates multiple pathologic remodeling processes in PAH (90). At least some of these effects in IL-6 transgenic mice are probably mediated by enhanced TGF-β signaling, as IL-6 has been demonstrated to augment TGF-β1 responses by reducing turnover of TGF-β receptors from the plasma membrane (94). The observation that BMPRII pathway signaling normally inhibits IL-6 expression in pulmonary vasculature suggests a potential mechanism by which *BMPR2* haploinsufficiency provides a vulnerable setting for runaway inflammatory and fibrotic signaling (95).

## Interplay Between Mechanobiology and TGF-β Superfamily Signaling in PAH

Biomechanical forces attributable to arterial physical properties and blood flow play prominent roles in vascular remodeling. Together with inflammatory signals, biomechanical forces and TGF-β superfamily signaling interact reciprocally during vascular homeostasis and disease initiation (96). Pathologic vascular remodeling in PAH is characterized both by narrowing of the distal pulmonary arterioles and resultant dilation of the larger, more proximal arteries. Changes in the width of the vessel lumen are accompanied by increased thickness and stiffness of vessel walls, properties that together reinforce the development of turbulent blood flow patterns throughout the pulmonary arterial tree (Figure 1). Turbulent flow itself in turn contributes to dysfunction and excessive proliferation of ECs, leading to neointimal lesions, and vascular occlusion (75). These structural, biomechanical, and proliferative changes could establish a positive feedback loop of pathologic vascular remodeling, especially around vascular branch points where turbulent flow forces are most pronounced. Indeed, neointimal lesions of proliferative ECs are found primarily at branch points (75). Furthermore, detailed temporal analysis in rodent PAH models reveals that arterial stiffening occurs early in the disease process, prior to hemodynamic changes and right ventricular dysfunction, suggesting that vessel wall stiffening is one determinant of disease onset (97).

Changes in the mechanical properties of pulmonary blood flow are interpreted by TGF-β superfamily receptors located in endothelial cells and affect changes in canonical superfamily signaling pathways. For example, steady-state laminar flow, the pattern typical of healthy vasculature, promotes EC quiescence by facilitating activation of the BMPRII-SMAD1/5/8 axis (Figure 1) (98). This signaling pathway prevents cell cycle progression by ECs and contributes to the stabilization of EC cellular junctions, preventing vascular remodeling processes. Laminar blood flow also promotes expression of the key BMPRII partner ALK1 (98) and promotes its association with the coreceptor endoglin, mechanisms that sensitize ECs to BMP9 signaling and aid in BMPRII pathway activation (99, 100). ALK1 therefore acts as a critical molecular link between blood flow and vascular quiescence (101). Furthermore, caveolin-1, itself a PAH risk factor that is regulated by shear stress forces, is required for proper membrane localization of BMPRII (102, 103). In elegant contrast with the quiescence-promoting role for the BMPRII pathway, disturbed flow patterns stimulate arterial remodeling through a mechanism dependent upon endothelial SMAD2/3 and ALK5 signaling (104, 105). These studies suggest an interesting model for the onset of PAH pathogenesis in which loss of BMPRII or one of its signaling partners removes a flow-regulated brake upon SMAD2/3-driven remodeling processes by pulmonary vascular ECs. Inflammation could enhance this pathogenic process by further diminishing BMPRII levels, and remodeling could beget further remodeling by disrupting laminar flow patterns important for BMPRII pathway activity (Figure 1).

TGF-β isoforms are prominent among superfamily ligands that require mechanical activation from a latent state to engage cognate receptors (8). Briefly, TGF-β isoforms are synthesized as inactive precursors consisting of a prodomain—referred to as the latency-associated peptide (LAP)—together with the mature ligand and are attached to extracellular matrix proteins through association with latent TGF-β binding proteins (LTBPs). Release of an active signaling domain from the inert TGF-β/LTBP complex depends upon the physical stiffness of the extracellular environment. Thus, it has long been hypothesized that pathologic TGF-β signaling in PAH and related fibrotic conditions operates through a positive feedback loop of extracellular matrix deposition, increased stiffness, and further TGF-β activation (106, 107). Proper sequestration of latent TGF-β complexes by LTBP proteins is known to be critical for the spatial and temporal regulation of TGF-β activation during homeostasis and disease (108) and might facilitate rapid signaling responses to physical insults. As discussed further below, the many types of proteins that control TGF-β localization and activity, including RGD-integrins, metalloproteinases, and thrombospondin-1, provide potential therapeutic targets for PAH treatment (109).

## Targeting Deficient SMAD1/5/8 Pathway Signaling

In the two decades since BMPRII deficiency was first implicated in the development of PAH, many approaches to promote SMAD1/5/8 pathway signaling in the pulmonary vasculature have been evaluated in PAH models and, in a few cases, clinically (Table 1).

**Table 1:** Agents targeting canonical TGF-β superfamily pathways for PAH.

**Pathway branch**	**Target**	**Agent and mechanism**	**Preclinical activity**	**Clinical evaluation**		
				**Study type**	**NCT ID**	**Status**
SMAD1/5/8	*BMPR2* gene	AdBMPR2 + Fab-9B9 or AdCMVBMPR2myc + Fab-9B9 (adenoviral delivery)	(110, 111)	–	–	–
	*BMPR2* promoter hypermethylation	Adenoviral delivery of *SIN3a*	(112)	–	–	–
	BMPRII	miR-20a inhibitor (antagomiR disinhibits BMPRII expression)	(113)	–	–	–
		6-Mercaptopurine (activation of Nur77)	(114)	–	–	–
		4-Phenylbutyrate (rescue of misfolded BMPRII)	(115)	–	–	–
		Ataluren/PTC124 (translational read-through of premature termination mutations)	(116)	–	–	–
	BMP9	Recombinant BMP9 (activation of BMPRII)	(117)	–	–	–
		Anti-BMP9 antibody (immunoneutralization)	(118)	–	–	–
	BMP2, BMP4, BMP7	Anti-gremlin1 antibody (disinhibition of specific BMPs)	(119)	–	–	–
	BMPRII-ALK1 signaling	FK506/tacrolimus (disinhibition of ALK1)	(120, 121)	Phase 2a	01647945	(122)
				Other	–	(123)
	Downstream target genes	Stabilized apelin analogs (activation of APJ)	(124, 125)	Other	01457170	(126)
		Nutlin-3 (rescue of p53-PPARγ complex)	(127)	–	–	–
SMAD2/3	ALK5	SD-208, SB-525334 (TKI)	(128, 129)	–	–	–
	ALK5, TGFBRII	Anti-TGF-β receptor antibody (immunoneutralization)	(64)	–	–	–
	TGF-β1, TGF-β2, TGF-β3	Pan anti-TGF-β antibody (multi-ligand sequestration)	(63)	–	–	–
	TGF-β1, TGF-β3	TGFBRII-Fc (multi-ligand sequestration)	(57)	–	–	–
	Latent TGF-β stabilization	LSKL peptide (competitive antagonism of thrombospondin-1)	(130)	–	–	–
	Activin-class ligands (activin A, GDF8, GDF11, activin B)	ActRIIA-Fc (multi-ligand sequestration)	(13)	Phase 2	03496207	(131)
				Phase 2a	03738150	Ongoing
				Phase 3	04576988	Ongoing
					04811092	Ongoing
					04896008	Ongoing

*APJ, apelin receptor; PPARγ, peroxisome proliferator-activated receptor gamma; TKI, tyrosine kinase inhibitor*.

### Restoration of BMPRII Expression

Preclinical studies have investigated delivery of the wild-type *BMPR2* gene by various methods to remedy BMPRII deficiency (110, 111, 132–134). These studies indicate that delivery of exogenous *BMPR2* to the pulmonary vascular endothelium can improve cardiopulmonary parameters in two different rodent models of PAH, in some cases on a preventive basis and in other cases therapeutically. As noted previously (15), two limitations of using viral vectors to deliver *BMPR2* to the endothelium are the transient nature of adenoviral transgene expression and the potential for deleterious mutations following genomic integration. Alternative methods of *BMPR2* delivery are therefore under investigation (134, 135). *BMPR2* gene delivery has not yet been studied clinically.

Epigenetic mechanisms, notably including hypermethylation of the *BMPR2* promoter, are implicated in PAH pathogenesis (136). The transcriptional regulator switch-independent 3a (*SIN3a*), recently found to be dysregulated in PAH patients and rodent models, promotes *BMPR2* expression in PASMCs through demethylation of its promoter (112). Increased BMPRII levels in these cells were accompanied by higher levels of pSMAD1/5/8, confirming activation of this pathway. Intratracheal delivery of *SIN3a* by adenoviral vector restored BMPRII expression, increased levels of pSMAD1/5/8, and improved cardiopulmonary endpoints in two rat models of PAH (112). This virally mediated approach to indirectly elevating *BMPR2* expression is associated with the same limitations as those noted above for direct *BMPR2* delivery. In addition, as it relies on the endogenous *BMPR2* gene, this approach is expected to be more effective in patients with reduced expression of wild-type *BMPR2* than in patients harboring *BMPR2* inactivating mutations.

Other diverse approaches for elevating BMPRII levels have yielded positive results in preclinical models of PAH. A promising approach involves activation of the orphan nuclear receptor Nur77, which is a key regulator of proliferation and inflammation in vascular cells. Treatment with 6-mercaptopurine increases expression of Nur77, BMPRII, pSMAD1/5/8, and target gene Id3 in pulmonary arterioles in a rat model of severe angioproliferative PAH (114). Moreover, therapeutic treatment with 6-mercaptopurine reversed abnormal vascular remodeling and RV hypertrophy in this model (114). In another approach, an antagonistic modified RNA oligonucleotide (antagomiR), which selectively targets the *BMPR2* negative regulator miR-20a, increased levels of BMPRII expression in lung tissue and improved cardiopulmonary parameters in a hypoxia-induced mouse model of PAH (113). A limitation of this study is that it did not evaluate therapeutic treatment in the context of established vascular pathology, which would better model the disease state in PAH patients with ongoing vascular remodeling. Other approaches have increased pulmonary expression of BMPRII in mice harboring certain *BMPR2* mutations either by rescuing misfolded BMPRII from the endoplasmic reticulum (115) or by facilitating translational read-through of premature *BMPR2* termination mutations (116). Such approaches provide support for future clinical evaluation in PAH patients with specific *BMPR2* mutations (116).

Of special note, the herbally-derived agent berberine was reported to improve cardiopulmonary endpoints in a hypoxia-induced mouse model by elevating expression of BMPRII and pSMAD1/5/8 while also reducing expression of TGF-β and pSMAD2/3 (137). Although investigation of target identity and further study are warranted, protective effects of berberine in this model underscore the potential benefit of rebalancing TGF-β superfamily signaling in PAH.

### Stimulation With Exogenous BMPs

The finding that exogenous BMP9 can reverse established disease in rodent models of PAH (117) suggested that a similar approach could be beneficial in patients. However, this strategy is controversial because subsequent preclinical studies indicate that the role of BMP9 in pulmonary vascular homeostasis is complex and likely context dependent (118, 138–140). Importantly, loss of BMPRII reverses the endothelial response to BMP9, paradoxically causing enhanced proliferation (138), and BMP9 promotes pulmonary vascular remodeling in mice under conditions of chronic hypoxia (139). Even if these conflicting aspects of BMP9 function were to be resolved in favor of a beneficial role in PAH, the clinical value of this approach would depend on development of BMP9 agonists with extended circulating half-lives to avoid impractical dosing regimens in patients.

### Disinhibition of Endogenous BMPs

BMP2, BMP4, and BMP7 exert anti-proliferative effects in pulmonary vessels through SMAD1/5/8 pathway activation. These ligands are selectively inhibited by the endogenous BMP antagonist gremlin-1, which is implicated as an important promoter of pathologic vascular remodeling in PAH (Figure 1) (141). Circulating levels of gremlin-1 stratify survival in PAH patients, hypoxia stimulates gremlin secretion by ECs, and *Grem1* haploinsufficiency reduces vascular remodeling in mice exposed to chronic hypoxia (41, 42). In addition, stretch-dependent secretion of gremlin-1 from pulmonary arterial cells is implicated in PAH induced by congenital systemic-to-pulmonary shunts and could potentially explain deficient BMPRII-pathway signaling in the many such patients whose *BMPR2* expression is normal (142). Importantly, therapeutic immunoneutralization of gremlin-1 reduces vascular remodeling in a mouse model of PAH (119). Despite its potential as a therapeutic candidate for PAH, anti–gremlin-1 antibody has not been evaluated clinically to our knowledge.

### Disinhibition of BMPRII/ALK1 Signaling

The immunosuppressive agent tacrolimus (FK506) has been evaluated more extensively than other activators of SMAD1/5/8 pathway signaling as a potential treatment for PAH. A large-scale screen of FDA-approved drugs identified FK506 as an effective BMPRII signaling activator that disinhibits ALK1 kinase activity through inhibition of the immunophilin FK-binding protein-12 (120). FK506 reversed dysfunctional BMPRII signaling in pulmonary ECs from patients with idiopathic PAH and reversed cardiopulmonary functional deficits and vascular remodeling when administered therapeutically in a rat model of severe angio-obliterative PAH (120). Based on these promising preclinical results, FK506 was assessed in a phase 2a tolerability and safety study in PAH patients (NCT01647945, *N* = 23), but the results were inconclusive (122) and could warrant follow-up evaluation in a larger patient population. Intriguingly, FK506 treatment at this dose led to substantial improvement and stabilization of cardiovascular function in three patients with end-stage PAH who did not qualify for the foregoing trial (123). Recent findings suggest that targeting the BMPRII pathway with FK506 may exert direct protective effects on the right ventricle independent of its beneficial effects on the pulmonary vasculature (121).

### Activation of Downstream Genes in Defective BMPRII Pathway

An alternative strategy to increase SMAD1/5/8 pathway activity is to promote expression of its downstream targets. One prominent target of BMPRII-mediated signaling in the endothelium is apelin (143), a peptide ligand of the apelin receptor whose activation opposes the renin-angiotensin-aldosterone system and regulates cardiovascular functions including hemodynamic homeostasis (144, 145). A genetic approach was used to identify apelin as a target gene of the BMP pathway in endothelial cells, and BMPRII-SMAD1/5/8 signaling was found to mediate downregulation of apelin expression by BMPs in such cells (146). However, the direction of this response is difficult to reconcile with later studies revealing apelin insufficiency in PAH lung and implicating apelin as beneficial in the context of PAH and cardiovascular disease more broadly. For example, circulating apelin levels are reduced in patients with PH, and apelin deficiency worsens hypoxia-induced PH in mice (147). Administration of a stabilized apelin analog, pyroglutamylated apelin-13, improves cardiopulmonary parameters in a monocrotaline rat model of PAH (124). In PAH patients undergoing right heart catheterization (NCT01457170, *N* = 19), this apelin analog reduced pulmonary vascular resistance and increased cardiac output without reducing mean pulmonary arterial pressure (126). Apelin receptor agonists with extended circulating half-lives are under development (125, 144), but substantial improvement will be needed for their use to become practical in a chronic clinical setting.

Additional agents have been evaluated preclinically as therapeutic activators of downstream targets in the SMAD1/5/8 pathway. One is nutlin-3, a small molecule which stabilizes a BMPRII-dependent transcription factor complex between p53 and PPARγ (peroxisome proliferator-activated receptor gamma) to activate a vasculoprotective gene regulation program downstream of BMPRII that includes the apelin gene (*APLN*) (127). This approach has been used to regenerate pulmonary microvessels and reverse persistent PH in mice with loss of BMPRII in pulmonary arterial ECs (127). Another therapeutic agent that can promote expression of BMPRII pathway effectors is tyrphostin-AG1296, a small-molecule tyrosine kinase inhibitor identified by screening compounds for improved survival of ECs from PAH patients (148). The tyrphostin-AG1296 mechanism of action remains to be defined but involves combined upregulation of BMPRII, SMAD1/5 coactivators, and cAMP response element-binding proteins, leading to an anti-PAH gene expression signature.

## Targeting Overactive SMAD2/3 Pathway Signaling

Inhibitors of the SMAD2/3 pathway have also been explored for treatment of PAH (Table 1) and could theoretically be used in combination with activators of the SMAD1/5/8 pathway as a strategy to rebalance superfamily signaling. As noted for berberine in the preceding section, some individual agents could potentially exert superfamily rebalancing effects through complementary actions on both SMAD pathways, either directly or indirectly through known mechanistic links between them.

### TGF-β

TGF-β inhibition was proposed more than a decade ago as a potential therapeutic approach to treat PAH (57, 63, 64, 128, 129, 149–151). Given the prominent roles of TGF-β in fibrotic diseases and cancer, diverse therapeutic approaches for inhibiting TGF-β-mediated signaling are being explored preclinically and clinically for those indications (152, 153) and could potentially be useful for treating PAH. Recent evidence indicates that there could be differential involvement of TGF-β-mediated signaling in patients with idiopathic PAH compared to those with hereditary PAH as well as differential involvement in rodent models of the disease (154).

### Inhibition of TGF-β Receptors

Small-molecule inhibitors of ALK5 display efficacy in rodent models of PAH (128, 129) but have not been evaluated in PAH patients due in part to safety concerns and the potential for off-target effects (155). Such tyrosine kinase inhibitors are not selective for ALK5, as they also inhibit the closely related ALK4 and ALK7 receptors, which mediate activin signaling (156). Additionally, ALK5 inhibition is not selective for TGF-β signaling because this type I receptor also mediates signaling by GDF11 (71, 72). An antibody capable of inhibiting both type I and type II receptors for TGF-β was reported to be efficacious in a monocrotaline rat model of PAH (64), but such receptor immunoneutralization has not been evaluated in patients with PAH.

### Inhibition of Active TGF-β Isoforms

More selective methods of TGF-β inhibition, including a pan anti–TGF-β antibody and a TGFBRII-Fc fusion protein that selectively sequesters TGF-β1 and TGF-β3, have displayed efficacy in rodent models of PAH (57, 63). Isoform selectivity in the latter case is thought to be advantageous partly due to major involvement of TGF-β2 in cardiac valve homeostasis and thus cardiotoxicity associated with ALK5 inhibition [see citations in ref. (57)]. These approaches for targeting TGF-β isoforms have not yet been evaluated clinically in PAH.

### Stabilization of Latent TGF-β

An intensely explored approach to inhibit TGF-β signaling involves stabilization of latent ligand normally sequestered in the extracellular matrix, thereby preventing release of TGF-β and its binding to receptors. Such activation is regulated endogenously by interaction of TGF-β-containing latent complexes with several types of proteins, including integrins, proteases, thrombospondin-1, and glycoprotein-A repetitions predominant protein (GARP) (157–159). Thrombospondin-1 is implicated preclinically in PH caused by either hypoxia or the parasite *Schistosoma mansoni*, and thrombospondin-1 inhibition by the synthetic tetrapeptide Leu-Ser-Lys-Leu protects mice from PH caused by either factor (130). Further reinforcing the vascular connection, thrombospondin-1 contributes to arterial stiffening caused by disturbed blood flow (160). Although not investigated in experimental PAH, integrin inhibitors and antibody-mediated stabilization of latent TGF-β display efficacy in models of fibrosis or cancer (161–164), providing preclinical support for such approaches generally. Because endogenous mechanisms of TGF-β activation vary depending on cellular and tissue context (158), the therapeutic effects produced by the foregoing approaches would be predicted to differ according to their respective targets. Moreover, such approaches targeting TGF-β regulation would likely produce a subset of the effects seen by directly targeting active TGF-β isoforms and therefore provide precision which could be advantageous in certain contexts.

### Inhibition of Activin-Class Ligands

Activin-class members of the SMAD2/3 signaling pathway have only recently been recognized widely as important contributors to PAH pathogenesis. Most prominent is the ligand trapping fusion protein ActRIIA-Fc, also known as sotatercept, which sequesters the activin-class ligands activin A, activin B, GDF8, and GDF11 with high affinity (165). As noted above, at least three of these ligands are upregulated in pulmonary vascular lesions of PAH patients and PAH rodent models (13). Protective administration of a murine ActRIIA-Fc fusion protein in rodent models markedly improves cardiopulmonary parameters and vascular remodeling, and therapeutic administration of this agent in a Sugen-hypoxia-normoxia model with established severe disease effectively alleviates PH and vascular remodeling (13). This activity is attributable in part to anti-proliferative effects on pulmonary arterial SMCs and ECs as well as to enhanced apoptosis in the vascular wall. Improvement in right ventricular structure and function may stem from indirect effects of reduced pulmonary vascular resistance and compliance. However, it could also arise from direct cardioprotective effects of ActRIIA-Fc consistent with those described previously with inhibition of activin receptor-mediated signaling in models of left ventricular failure associated with aging or systemic pressure overload (166, 167).

A phase 2 study of ActRIIA-Fc has been conducted in patients with PAH receiving background therapy (131). In this study (NCT03496207, *N* = 106), sotatercept produced significant improvement in the primary endpoint, pulmonary vascular resistance. It was also associated with clinically meaningful improvements in 6-min walk distance and circulating levels of N-terminal pro-B-type natriuretic peptide, a marker for cardiac dysfunction. Sotatercept has previously been evaluated in healthy volunteers and patients with conditions characterized by dysfunctional TGF-β superfamily signaling, including anemia associated with myelodysplastic syndromes, anemia associated with β-thalassemia, chemotherapy-induced anemia, end-stage kidney disease, bone loss, and multiple myeloma (168–174). As a result, substantial data are already available regarding sotatercept's safety profile. Ongoing clinical studies of sotatercept in PAH patients include a phase 2 study for detailed characterization of cardiopulmonary status by right heart catheterization with exercise (NCT03738150) and several phase 3 registration-enabling studies (NCT04576988, NCT04811092, NCT04896008). Interestingly, several clinical studies have determined that sotatercept increases circulating hemoglobin concentrations under diverse conditions (169, 171–174). It has been observed that a large proportion of PAH patients exhibits anemia and could therefore receive sotatercept with acceptable erythropoietic effects (175). Further study is required to determine the potential benefits of increasing hemoglobin levels concurrently with targeting cardiopulmonary remodeling in PAH patients, particularly those with anemia.

## Conclusions

Our growing understanding of mechanisms responsible for initiation and progression of PAH has not yet been matched by development of therapies effectively targeting those underlying disease processes. The TGF-β superfamily of ligands and receptors plays a critical role in the development and severity of PAH. More precisely, an imbalance in the intracellular SMAD2/3 vs. SMAD1/5/8 signaling pathways is now widely accepted to be an important contributor. Therapies targeting these two SMAD pathway branches have been evaluated preclinically and are in a few cases in clinical development. Importantly, there are indications that therapeutic interventions targeting the TGF-β superfamily have the potential to be disease modifying. Indeed, preclinical and clinical data generated with an ActRIIA-Fc ligand trap in particular support the view that targeting cellular proliferation through a rebalancing of SMAD activation could be a beneficial therapeutic approach for the underserved PAH patient population.

Further work is needed to establish whether other therapeutic modalities such as those targeting mechanisms of latent ligand activation could be developed to either reduce pro-proliferative or enhance anti-proliferative TGF-β superfamily pathways. Other interesting but underexplored possibilities are that either concomitant modulation of SMAD2/3 and SMAD1/5/8 pathways or combined inhibition of SMAD2/3 pathway-activating ligands (activin-class ligands together with one or more TGF-βs) could provide benefits beyond those observed to date with more restricted approaches. Such combinatorial strategies might also be useful in the context of Group 3 PH, a disease category characterized by pulmonary vasculopathy with fibrotic lung disease, most notably in subgroups associated with interstitial lung disease (PH-ILD) or chronic obstructive pulmonary disease (PH-COPD). In addition, it remains to be determined whether precisely targeting a single intracellular TGF-β superfamily pathway, including canonical and non-canonical effectors, could offer a different therapeutic window or greater ease of use than inhibition of ligand-receptor interactions. Hence, two decades after the seminal observations linking familial PAH with TGF-β superfamily signaling, there are signs that targeting this pathway—now thought to control multiple PAH disease processes—could offer hope for transformative PAH treatments.

## Author Contributions

PA, SB, and MA drafted the manuscript. PA, SJ, SB, MA, GL, and RK revised the manuscript critically for intellectual content. All authors contributed to the article and approved the submitted version.

## Funding

The authors were supported by funding provided by Acceleron Pharma.

## Conflict of Interest

All authors are past employees of Acceleron Pharma and are now employees of Merck Sharp and Dohme Corp., a subsidiary of Merck & Co., Inc., Kenilworth, NJ, USA and may own stock and hold stock options in the Company.

## Publisher's Note

All claims expressed in this article are solely those of the authors and do not necessarily represent those of their affiliated organizations, or those of the publisher, the editors and the reviewers. Any product that may be evaluated in this article, or claim that may be made by its manufacturer, is not guaranteed or endorsed by the publisher.
